# Potential developmental neurotoxicity of pesticides used in Europe

**DOI:** 10.1186/1476-069X-7-50

**Published:** 2008-10-22

**Authors:** Marina Bjørling-Poulsen, Helle Raun Andersen, Philippe Grandjean

**Affiliations:** 1 Department of Environmental Medicine, University of Southern Denmark, Winslowparken 17, 5000 Odense, Denmark; 2 Department of Environmental Health, Harvard School of Public Health, Landmark Building 3E-110, 401 Park Drive, Boston, MA 02215, USA

## Abstract

Pesticides used in agriculture are designed to protect crops against unwanted species, such as weeds, insects, and fungus. Many compounds target the nervous system of insect pests. Because of the similarity in brain biochemistry, such pesticides may also be neurotoxic to humans. Concerns have been raised that the developing brain may be particularly vulnerable to adverse effects of neurotoxic pesticides. Current requirements for safety testing do not include developmental neurotoxicity. We therefore undertook a systematic evaluation of published evidence on neurotoxicity of pesticides in current use, with specific emphasis on risks during early development. Epidemiologic studies show associations with neurodevelopmental deficits, but mainly deal with mixed exposures to pesticides. Laboratory experimental studies using model compounds suggest that many pesticides currently used in Europe – including organophosphates, carbamates, pyrethroids, ethylenebisdithiocarbamates, and chlorophenoxy herbicides – can cause neurodevelopmental toxicity. Adverse effects on brain development can be severe and irreversible. Prevention should therefore be a public health priority. The occurrence of residues in food and other types of human exposures should be prevented with regard to the pesticide groups that are known to be neurotoxic. For other substances, given their widespread use and the unique vulnerability of the developing brain, the general lack of data on developmental neurotoxicity calls for investment in targeted research. While awaiting more definite evidence, existing uncertainties should be considered in light of the need for precautionary action to protect brain development.

## Introduction

Pesticides are used widely in agriculture to maintain and increase crop yields, and they are also applied in homes and gardens. The annual application of synthetic pesticides to food crops in the EU exceeds 140,000 tonnes [1], an amount that corresponds to 280 grams per EU citizen per year. Despite European policies to reduce pesticide use, EU statistics data for 1992–2003 show that the annual pesticide consumption has not decreased [1]. A few hundred different compounds are authorised for use in all EU member states, but a similar number of pesticides is in current use in different EU countries and are being evaluated for possible authorisation in all of EU. Approximately 300 different pesticides have been reported as contaminants of food products of European origin [2]. Up to 50 percent of fruits, vegetables and cereals grown in the European Union are known to contain pesticide residues [2], but only a small fraction of pesticides in current use are included in the monitoring programmes. Nonetheless, one out of twenty food items is known to exceed a current EU legal limit for an individual pesticide [2]. Further, over 25% of fruits, vegetables, and cereals contain detectable residues of at least two pesticides [2]. Processed food and baby food are also commonly contaminated. In addition, other sources, such as contaminated drinking water, dusts and spray drift contribute to human exposures.

The total level of population exposures to pesticides in Europe is unknown, but data from US population studies show that the majority of the population has detectable concentrations of methyl phosphate, ethyl phosphate, and other pesticide metabolites in the urine [3].

Many pesticides target the nervous system of insect pests. Because of the similarity of neurochemical processes, these compounds are also likely to be neurotoxic to humans. This concern is of particular relevance to the developing human brain, which is inherently much more vulnerable to injury caused by toxic agents than the brain of adults [4]. During prenatal life, the human brain must develop from the ectodermal cells of the embryo into a complex organ consisting of billions of precisely located, highly interconnected, and specialised cells. For optimum brain development neurons must move along precise pathways from their points of origin to their assigned locations, they must establish connections with other cells, and they must learn to communicate with other cells via these connections [4-6]. All of these processes have to take place within a tightly controlled time frame, and each developmental stage has to be reached on schedule and in the correct sequence. If a developmental process in the brain is halted or inhibited, there is little potential for later repair, and the consequences may therefore be permanent [4,6].

Concerns in regard to developmental neurotoxicity due to pesticides have been fuelled by recent epidemiologic observations that children exposed prenatally or during early postnatal life suffer from various neurological deficits [7-12]. Urinary pesticide metabolite concentrations associated with adverse effects overlap with the ranges that occur in the general population [3]. Although the identity of the parent pesticides and the exact magnitude of causative exposures are unclear, these observations suggest that developmental neurotoxicity from pesticide exposure is a public health concern.

Despite the increasing recognition of the need to evaluate developmental neurotoxicity in safety assessment [13-15], only very few of the commercial chemicals in current use have been examined with respect to neurodevelopmental effects [16]. Validated rodent models exist, but they are considered expensive and are only infrequently used. According to the current EU Plant Protection Directive (91-414-EEC), a neurotoxicity test in hens is required only for organophosphates and some carbamates to assess the possible risk of delayed peripheral neurotoxicity following acute exposure.

From a public health viewpoint, the prevention of neurodevelopmental disorders is a priority; these disorders include learning disabilities, attention deficit hyperactivity disorder (ADHD), autism spectrum disorders, developmental delays, and emotional and behavioural problems. The causes of these disorders are unclear, and interacting genetic, environmental, and social factors are likely determinants of abnormal brain development [17]. Medical statistics data are difficult to compare between countries, but one report suggests that 17% of US children under18 years of age suffer from a developmental disability, in most cases affecting the nervous system [18]. In calculations of environmental burdens of disease in children, lead neurotoxicity to the developing brain is a major contributor [19]. Pesticide effects could well be of the same magnitude, or larger, depending on the exposure levels.

A recent review [16] listed 201 chemicals known to be neurotoxic in humans; only 5 of these substances have been firmly documented as causes of developmental neurotoxicity. Identification of human neurotoxicity was based on available evidence, including poisoning incidents described in the scientific literature, as identified from the Hazardous Substances Data Bank of the U.S. National Library of Medicine. Although published clinical information may not be representative for the relative neurotoxicity risks due to industrial chemicals, it is noteworthy that a total of 90 (45%) of the neurotoxic substances are pesticides. For these substances, only neurotoxicity in adults had been documented, thereby documenting that access to the brain is possible and may cause toxic effects. Given the vulnerability of the developing brain, it is likely that many of these substances will also be capable of causing developmental neurotoxicity [16]. Indeed, studies in laboratory animals support the notion that a wide range of industrial chemicals can cause developmental neurotoxicity even at low doses that are not harmful to mature animals [14,20].

Given the likely importance of pesticides in regard to developmental neurotoxicity in humans, this review focuses on pesticides approved for current use in Europe, i.e. either authorised or being evaluated for authorisation within the European Union (Table 1). Our literature search was conducted by similar means as the previous review mentioned above [16], but included relevant data from laboratory experiments. The pesticides are grouped in accordance with the likely mechanism of action or chemical similarity. We focus on substances with a primary application as pesticides and therefore exclude substances like nicotine, warfarin, and ethanol with other primary uses.

**Table 1:** Neurotoxic pesticides, which are authorised or pending evaluation for authorisation in the EU

**Pesticide**	**Annex 1 status**
**Organophosphate insecticides**	
Chlorpyrifos	In
Dimethoate	In
Ethoprophos	In
Phosmet	In
Fenamiphos (nematicide)	In

**Carbamates**	
Pirimicarb	In
Methomyl	Application resubmitted

**Pyrethroid insecticides**	
Cypermethrin (type II)	In
Deltamethrin (type II)	In
Pyrethrum/pyrethrin (natural pyrethrin)	Pending

**Other insecticides**	
Nicotine	Pending

**Dithiocarbamate fungicides**	
Maneb	In
Thiram	In

**Chlorophenoxy herbicides**	
2,4-D	In

**Bipyridyl herbicides**	
Diquat dibromide	In

**Rodenticides**	
Warfarin	In

**Fumigants**	
Phosphides (zinc, magnesium, and aluminum phosphides)	Pending
Sulfuryl fluoride	Pending

The list includes pesticicides, which are registered as "in" or "pending" on the current EU Annex 1 list (as of August 2008), and for which neurotoxicity in humans has been reported in The Hazardous Substances Data Bank and/or the NIOSH Pocket Guide to Chemical Hazards. The full Annex 1 list with the status of active substances under EU review can be downloaded as an Excel sheet at .

### Search strategy and selection

We first identified pesticides that have caused neurotoxic effects in humans from the Hazardous Substances Data Bank (HSDB) of the U.S. National Library of Medicine [16]. We searched for the terms "pesticide" and "neuro*". From the list of substances obtained in this way, we identified the pesticides, for which neurotoxic effects in humans had been reported. In addition, we searched the U.S. National Institute of Occupational Safety and Health (NIOSH) – Pocket Guide to Chemical Hazards , using the search terms "pesticide", "insecticide", "herbicide", "fungicide", "fumigant", and "rodenticide" in combination with "central nervous system". The list of neurotoxic pesticides identified in this way was then compared to the current Annex 1 list (as of August, 2008) of pesticides authorised in the European Union according to Plant Protection Directive 91-414-EEC (an Excel data sheet with the status of active substances under EU review can be downloaded from ). We chose pesticides with an Annex 1 status "in" or "pending" for consideration (Table 1).

For each neurotoxic pesticide in current use, we searched PubMed to identify published data on developmental neurotoxicity. We used pesticide synonyms, commercial names and the CAS number, in combination with each of the terms "neurotoxic", "neurotoxicity", "neurologic", "neurological" and "nervous system", and additional searches included the terms "prenatal", "pregnancy", "fetus", "fetal", "maternal", "developmental" and "child".

### Organophosphate insecticides

#### Toxic mechanisms

The primary target of organophosphate (OP) insecticides is the enzyme acetylcholinesterase (AChE), which hydrolyses the neurotransmitter acetylcholine in both the peripheral and the central nervous system. OPs containing a P = O moiety are effective inhibitors of AChE, whereas OPs with a P = S moiety require bioactivation to form an "oxon" or oxygen analogue of the parent compound. Inhibition of AChE by OPs is obtained by the P = O moiety forming a covalent bond with the active site of the enzyme. The enzyme-inhibitor complex can become "aged" by a non-enzymatic hydrolysis of one of the two radical groups in the OP, and once the complex has aged, inhibition of AChE is irreversible (reviewed in [21]). Inhibition of AChE causes accumulation of acetylcholine at cholinergic synapses, leading to over-stimulation of muscarinic and nicotinic receptors. In addition, acetylcholine has important functions during brain development [22].

In severe cases of OP poisoning in adults (AChE inhibition exceeding 70%) [23], a "cholinergic syndrome" is elicited, including various central nervous system (CNS) effects such as headache, drowsiness, dizziness, confusion, blurred vision, slurred speech, ataxia, coma, convulsions and block of respiratory centre [24]. Some OPs can also induce a delayed neuropathy which does not involve inhibition of AChE but rather the neuropathy target esterase (NTE) [25,26]. The physiological functions of NTE are still unknown, and it is obscure how phosphorylation and aging of NTE leads to axonal degeneration [27].

The syndromes described above are observed only following high dose, acute exposures to OPs. Survivors recover from these syndromes, but it is likely that the exposure also produces long-term adverse health effects. In rats, a single high exposure to an OP can cause long lasting behavioural effects [28,29], and the same has been reported from several human studies (e.g. [30,31]).

The concern is growing that also chronic, low exposures to OPs may produce neurological effects, although the evidence remains somewhat equivocal (reviewed in [32-34]). Most studies have found an association of OP exposure with increased neurological symptom prevalence. As an example, Hispanic immigrant farm workers in the US have a poorer neurobehavioural performance than non-agricultural Hispanic immigrants. Within the group of agricultural workers there was a positive correlation between urinary OP metabolite levels and poorer performance on some neurobehavioural tests [35]. A cross-sectional study of pesticide applicators reported that neurological symptoms were associated with cumulative exposure to moderate levels of organophosphate and organochlorine insecticides, regardless of recent exposure history [36].

Acetylcholine and other neurotransmitters play unique trophic roles in the development of the CNS [37,38], and inhibition of AChE by OPs and the resulting accumulation of acetylcholine may then conceivably disturb this development. Still, developing rats recover faster from AChE inhibition than adults, largely due to the fact that developing organisms have a rapid synthesis of new AChE molecules [39-41]. It therefore seems that either developmental toxicity may be unrelated to AChE inhibition, or that even a brief period of AChE inhibition is sufficient to disrupt development [42].

Chlorpyrifos is the most extensively studied OP with respect to developmental neurotoxicity in laboratory models. Prenatal or neonatal exposure has been shown to cause a variety of behavioural abnormalities in both mice and rats, including changes in locomotor skills and cognitive performance [43-46]. At concentrations comparable to those found in human meconium [47], experiments on rat embryo cultures showed mitotic abnormalities and evidence of apoptosis during the neural tube development stage, and significant effects even at concentrations more than an order of magnitude below those present in human meconium [48]. However, exposure of rat foetuses to chlorpyrifos by maternal administration did not induce large immediate effects on brain development [49], but chlorpyrifos treatment during gestation, did cause deficits in brain cell numbers, neuritic projections, and synaptic communication, which emerged in adolescence and continued into adulthood. This finding indicates that chlorpyrifos exposure during gestation results in altered programming of synaptic development [50,51].

The window of vulnerability to chlorpyrifos extends into relatively late stages of brain development, and chlorpyrifos can induce neurobehavioural abnormalities during the second and third postnatal weeks in rat [43,52,53], corresponding to the neonatal stage in humans [54]. This period is outside the major phase of neurogenesis in most brain regions, but it is the period of peak gliogenesis and synaptogenesis; developing glia have been found to be even more sensitive to chlorpyrifos than neurons [55-57].

Deficits elicited by prenatal exposure to chlorpyrifos are evident even at exposures below the threshold for detectable AChE inhibition, i.e. far below the 70% inhibition of AChE required for systemic toxicity in adults [43-46,51]. These findings suggest that mechanisms other than inhibition of AChE activity may, at least in part, be responsible for the developmental neurotoxicity of chlorpyrifos.

The non-cholinergic mechanisms of chlorpyrifos are not clear, but a possible target may be the signalling cascades involved in neuronal and hormonal inputs, including the cyclic-AMP – protein kinase A cascade, receptor signalling through protein kinase C, and direct effects on the expression and function of nuclear transcription factors mediating the switch from proliferation to differentiation, including c-fos, p53, AP-1, Sp1 and CREB (Ca^2+^/cAMP response element binding protein) (reviewed in [42]).

The notion that chlorpyrifos may exert developmental neurotoxicity through mechanisms other than inhibition of AChE opens the possibility that OPs may have compound specific effects that may be unrelated to the common AChE inhibitory effect. For example, microarray analysis has shown that the two OPs, chlorpyrifos and diazinon, have many similar effects on gene expression in the neonatal rat brain, but also notable disparities. All of the changes in gene expression induced by the two OPs were observed with doses, which did not induce biologically significant AChE inhibition [58,59]. In neonatal rats, diazinon and chlorpyrifos elicit each their unique pattern of damage/repair and altered synaptic function, even though OPs as a class target neural cell development and ACh systems [60].

Thus, findings of OP induced developmental neurotoxicity through individual mechanism other than the common AChE inhibition complicate extrapolation of effects from one OP to another. The existence of clear effects of OPs at doses below the threshold for AChE inhibition clearly demonstrate that it is inadequate to use AChE measurements alone as a biomarker for defining safe exposure limits for developmental neurotoxicity of OPs [60].

#### Epidemiologic evidence

With respect to developmental neurotoxicity of OPs in humans, knowledge is still relatively sparse, and most studies reflect exposures to more than one pesticide.

In California, USA, an association was found between reflex abnormalities in neonates and increased concentrations of OP metabolites measured in the mother's urine during pregnancy [7]. In a follow-up of the same cohort, urinary dialkyl phosphate metabolite levels during pregnancy, particularly from dimethyl phosphate pesticides, were negatively associated with mental development in the children at 24 months of age. No associations were observed between neurodevelopment and metabolites specific to malathion and chlorpyrifos [8].

In a cohort study of mothers and infants in New York City, USA, maternal levels of chlorpyrifos above the limit of detection, coupled with low maternal levels of paraoxonase activity (an enzyme which hydrolyses certain OPs, including chlorpyrifos oxon), were associated with reduced head circumference in the infants [61]. In the same cohort, prenatal levels of OP metabolites in the mother's urine were associated with anomalies of primitive reflexes in the infants [9].

In another New York City cohort, prenatal chlorpyrifos exposures were found to be inversely associated with birth weight and length [62]. In a follow up of this study, the children's cognitive and motor development was examined at 1, 2 and 3 years of age. The adjusted mean 3-year Psychomotor Development Index and Mental Development Index scores of the highly exposed children differed by 7.1 and 3.0 points, respectively, from the scores of children with low prenatal exposure to chlorpyrifos. The proportion of delayed children in the high-exposure group, compared with the low-exposure group, was five times greater for the Psychomotor Development Index and 2.4 times greater for the Mental Development Index [10].

Ecuadorian schoolchildren, whose mothers had been exposed to OPs and other pesticides during pregnancy by working in greenhouses, showed visuospatial deficits compared to children, whose mothers had not been exposed to pesticides during pregnancy. Furthermore, current exposure of the children, measured as the excretion of OP metabolites in urine, was found to be associated with increased reaction time [11].

In two US states, Ohio and Mississippi, children were acutely exposed to the OP, methyl parathion, and when analysed for neurobehavioural development, the exposed children were found to suffer from persistent problems with short-term memory and attention [12].

Although the epidemiological evidence for developmental neurotoxicity of OPs in humans is relatively sparse, there are clear indices of adverse effects. Urinary pesticide metabolite levels in the above studies were similar to those that have been recorded from the US general population [3,63] and in EU countries [64-66].

### Carbamate insecticides

Carbamate insecticides, like the OP insecticides, inhibit AChE and elicit cholinergic hyperstimulation. However, carbamates cause only reversible inhibition of AChE [67]. Thus, AChE inhibition by carbamates lasts only minutes or hours, whereas the effects of OPs with respect to AChE can last for 3–4 months (reviewed in [32]). Because of the transient inhibition of AChE, acute intoxication by carbamates generally resolves within a few hours [67].

When comparing the clinical course of carbamate poisoning (by aldicarb or methomyl) in young children (1–8 years old) and adults (17–41 years old), it was found that the predominant symptoms in children were CNS depression and hypotonia, and the most common muscarinic effect was diarrhoea. In adults the main symptoms were miosis and fasciculations, whereas CNS depression, hypotonia, and diarrhoea were uncommon [68]. Symptoms in children poisoned with OPs were found to be similar to symptoms for carbamate poisoning [69]. Thus symptoms of carbamate poisoning do not differ markedly from symptoms of OP poisoning in children, but rather the symptoms in children, differ from symptoms in adults.

It is possible that some carbamates may also be involved in oxidative stress [70,71]. The carbamate, carbofuran, has been observed to accentuate oxidative stress in rat brain by inducing lipid peroxidation and diminishing the antioxidant defence [70].

As for the OPs, it is likely that poisoning with carbamates may result in long term neurological effects [72]. Two patients showed cognitive deficit in attention, memory, perceptual, and motor domains 12 months after a poisoning incident [72]. With respect to long term, low level exposures to carbamates, reports concerning chronic toxicity are almost non-existent.

No epidemiological studies of developmental neurotoxicity of carbamates in humans could be found, and data from animal experiments are very sparse as well.

Assuming that some of the neurotoxic effects observed in association with prenatal exposure to OPs, such as chlorpyrifos, are due to inhibition of AChE, it is possible that carbamates may have similar developmental effects, even though the inhibition of AChE by carbamates is only transient. Induction of oxidative stress by some carbamates might also cause developmental neurotoxicity. It should also be noted that the carbamate physostigmine inhibits DNA synthesis in undifferentiated neuronotypic PC12 cells (a standard *in vitro *model for neuronal development). When differentiation was induced, adverse effects on DNA synthesis were intensified, and effects on cell number after prolonged exposure were also worsened by differentiation. Furthermore, differentiating cells displayed signs of oxidative stress, as measured by lipid peroxidation. Finally, the transmitter fate of the cells was shifted away from cholinergic phaenotype toward the catecholaminergic phaenotype. Similar findings were made when incubating the cells with the OPs chlorpyrifos, diazinon and parathion [73].

### Pyrethroid insecticides

The pyrethroids are a class of insecticides derived from naturally occurring pyrethrins from the *Chrysanthemum *genus of plants [74]. Pyrethroids contain several common features: an acid moiety, a central ester, and an alcohol moiety. Several stereoisomers exist of each pyrethroid compound, and their effects are stereospecific, indicating presence of specific binding sites (reviewed in [75]).

The acute toxicity of pyrethroids is mainly mediated by prolongation of the kinetics of voltage-gated sodium channels, which are responsible for generation of the inward sodium current that produces the action potential in excitable cells. Specific interaction of pyrethroids with the sodium channel slows down both the activation and inactivation properties of the channel, leading to a hyperexcitable state. Although activation is slowed at the single channel level, the density of sodium channels in excitable cells is so high that there are always sufficient unmodified channels to ensure that the activation phase of the action potential is not delayed. However, in the falling phase of the action potential, even a low proportion of modified channels can generate enough extra current to delay inactivation. This delay causes prolonged depolarisation, which, if the current is large enough and lasts long enough for neighbouring unmodified channels to recover excitability, can trigger a second action potential (reviewed in [76]).

Two types of pyrethroid structures exist. The type II pyrethroids contain a cyano-group in the α-position, whereas type I pyrethroids do not contain a cyano-group [77]. The two types differ with respect to the toxic signs they produce in rats, and with respect to the prolongation time of the sodium current they induce. Type I compounds prolong channel opening just long enough to induce repetitive firing of action potentials (time constants less than 10 msec), whereas type II compounds (time constants of more than 10 msec) hold the channels open for so long that the membrane potential ultimately becomes depolarised to the point at which generation of action potentials is no longer possible (reviewed in [75]).

Human pyrethroid poisoning is rare, and almost entirely involves type II pyrethroids. The main adverse effect of dermal exposure to type II pyrethroids is paresthesias, presumably due to hyperactivity of cutaneous sensory nerve fibres. Dizziness, headache and fatigue are common symptoms following ingestion of type II pyrethroids. In severe cases coma and convulsions are the principal life-threatening features [77].

The effects of pyrethroids on the CNS are complex and may also involve antagonism of γ-aminobutyric acid (GABA), modulation of nicotinic cholinergic transmission, enhancement of noradrenalin release, and direct actions on calcium or chloride ion channels. Still, because neurotransmitter-specific pharmacological agents do not protect very well against pyrethroid poisoning, it is unlikely that any one of these effects represents a primary toxic mechanism of action of pyrethroids. More likely, they are secondary to the effects on sodium channels, since most neurotransmitters are released secondary to increased sodium entry (reviewed in [76]).

In the few existing accounts of poisonings of adults with pyrethroids, successful recovery after the acute phase of poisoning has been described [78,79]. However, no detailed neuropsychological testing was applied to these patients, and also no *post mortem *examinations have been reported, and therefore it is unknown if such poisonings may have lasting effects. Likewise, no information is available on long term effects of low level chronic exposure in humans.

Neonatal rats are 4–17 times more vulnerable to the acute toxicity of pyrethroids (including permethrin (type I), deltamethrin (type II), cypermethrin (type II)) than adult rats [80,81]. The higher toxicity in neonates is affected by the lower capacity for metabolic detoxification, since neonates and adults have similar brain concentrations at different, but equitoxic, doses [80]. However, another study did not observe any age-dependency of the toxicity of the two type I pyrethroids, cismethrin and permethrin [82]. It has therefore been argued that age-dependent sensitivity to pyrethroids is only apparent at high acute doses, not at doses below those causing overt toxicity [82].

In addition to the possibility that young animals are more vulnerable to pyrethroids due to lower metabolic detoxification, there is also a possibility that increased vulnerability in young animals may be due to more specific effects of early life exposures. For example, several studies have found that embryonically expressed forms of voltage-gated sodium channels are replaced by adult forms as neurodevelopment proceeds (reviewed in[75]), and this difference in expression profile may affect the sensitivity towards pyrethroids. In mutation and knockout models of the voltage-gated sodium channels, perturbation of channel function during development impairs nervous system structure and function, underlining the importance of these channels in neurodevelopment. (reviewed in [75]).

Also in humans, perturbations of nervous system development have been associated with altered structure and function of voltage-gated sodium channels. Mutations in genes encoding sodium channel subunits have been identified, which result in neuronal hyperexcitability due to subtle changes in channel gating and inactivation [83]. Since pyrethroids also alter the activation and inactivation of sodium channels, and thereby the neuronal excitability, it is possible that these may have effects similar to mutations in the sodium channels. However, the mechanisms and magnitude of mutational versus pyrethroid effects are different, and also the duration of effect will differ (pyrethroids have a relatively short half-life, whereas mutations are permanent) [75].

Another possible indication that pyrethroid effects on sodium channels may be relevant to neurodevelopment is the observation that developmental exposure to phenytoin, an anticonvulsant that blocks sodium channels and other ion channels, disrupts nervous system structure and function [84]. The use of anticonvulsants during pregnancy has been associated with adverse effects, including microcephaly and intellectual impairment (reviewed in [75]). Although differences in doses and in pathogenesis may occur, this evidence would support a concern about the effect of pyrethroids on ion channels.

All existing studies of developmental neurotoxicity of pyrethroids were conducted with rodents as test animals, and although several of them have reported persistent changes in behaviour and/or neurochemistry in the animals, results appear somewhat inconsistent (reviewed in [75]). Several studies performed by Eriksson's group [85-87] have shown that mice exposed to pyrethroids on postnatal day 10–16 exhibit increased motor activity and a lack of habituation. These mice exhibit changes in density of muscarinic acetylcholine receptor (mAChR) binding for as long as 5 months after cessation of exposure [88]. Others have reported persistent changes in behaviour and/or biochemistry, including learning [89], motor activity [90], sexual behaviour [91], mAChR expression [92,93], and blood-brain barrier permeability [94]. A recent study in rats showed that neonatal exposure to permethrin and cypermethrin caused lasting behavioural effects, changes in monoamine concentrations in the striatum as well as increased oxidative stress [95]. In one study, both male and female mice were exposed to the type I pyrethroid, permethrin, before mating, and the following functions were affected in the offspring (with parental exposure to 9.8 mg/kg/day or more for 4 weeks before mating): development of reflexes, swimming ability and open field activity [96].

The potential developmental neurotoxicity of pyrethroids has also been investigated *in vitro *using cell lines. For example non-toxic concentrations (10^-6 ^M) of bifenthrin inhibited neurite outgrowth in PC12 cells, indicating that bifenthrin may have deleterious effects on the developing nervous system at concentrations lower than those capable of causing toxicity in the adult brain [97].

Existing data indicate that human exposures to pyrethroids occur and result in detectable concentrations in body fluids [98-100], but there is insufficient information available to adequately evaluate the range of internal doses in humans, and the consequences of these exposures are so far unknown.

### Dithiocarbamate fungicides

Dithiocarbamates are non-cholinesterase inhibiting, sulfur-containing carbamates, which are primarily used as fungicides and herbicides. Four major classes of dithiocarbamates exist; the methyldithiocarbamates, the dimethyldithiocarbamates, the diethyldithiocarbamates (DEDC), and the ethylenebisdithiocarbamates (EBDCs) (reviewed in [101]). The dithiocarbamates used as fungicides include metam sodium (methyldithiocarbamate), thiram (dimethyldithiocarbamate/tetramethyldithiocarbamate), and several EBDCs (mancozeb, maneb, metiram, zineb and nabam).

Dithiocarbamates form lipophilic complexes with di- and trivalent metallic cations, bonding through the sulfur atoms [102]. They are non-specific in action, and it is difficult to identify a single mechanism for their neurotoxic effects. Because of their metal-chelating capacity and their affinity for sulfhydryl groups, they are biologically highly active [103,104]. DEDCs are particularly known to modify the cellular redox state by inducing a copper-dependent oxidative stress [105,106], and inhibition of cytosolic Cu/Zn superoxidedismutase (SOD1), a key enzyme in the antioxidant response, has been observed in mice treated with DEDC [107]. The EBDCs can uncouple the mitochondrial electron transport chain [108,109]. Mitochondrial dysfunction is often associated with generation of reactive oxygen species (ROS), and ROS production was also found to play a role in mancozeb induced neuronal toxicity in mesencephalic cells, likely via redox cycling with extracellular and intracellular oxidases [110]. Further, ethylenethiourea (ETU), which is a degradation product of EBDCs, has been shown to inhibit thyroid peroxidase (TPX), the enzyme that catalyses synthesis of the thyroid hormones [111,112]. In addition, interference of dithiocarbamates with the vesicular transport of glutamate may play a role in their neurotoxicity [113]. Due to the differences in biochemical effects, these compounds seem to exhibit a range of different potencies in regard to developmental neurotoxicity.

Dithiocarbamates are reported to display low acute toxicity in humans and experimental animals [114]. Both in humans and laboratory animals, prolonged exposure to dithiocarbamates may cause neurotoxicity. Notably, peripheral neuropathy and extrapyramidal symptoms resembling parkinsonism have been associated with chronic exposure to dithiocarbamate pesticides [115].

As mentioned, chronic exposure of humans to EBDCs has been associated with neurocognitive impairment and parkinsonism [116]. In particular, exposure to maneb, which contains manganese, has been linked to development of parkinsonian-like symptoms in agricultural workers [117,118]. This finding may be related to the inhibition of complex III of the mitochondrial electron transport chain [108], disruption of the glutathione antioxidant system in dopaminergic cells [119], inhibition of proteasomal function and induction of α-synuclein aggregates in dopaminergic cells [120], induction of catechol autooxidation [121], and potentiation of the neurotoxicity of 1-methyl-4-phenyl-1,2,3,6-tetrahydropyridine (MPTP) in mice [122-124]. All of these observations support the notion that maneb may cause parkinsonian-like symptoms. DEDCs, though not methyldithiocarbamate, can also enhance MPTP- induced striatal dopamine depletion in mice [124].

Both thiram and ziram (dimethyldithiocarbamates) can induce apoptotic cell death in PC12 cells, in a dose- and time-dependent manner [125]. Both compounds induced rapid and sustained increases of intracellular Ca^2+ ^in the cells, which were almost completely blocked by flufenamic acid, an inhibitor of non-selective cation channels. Also, BAPTA-AM, which is an intracellular Ca^2+ ^chelator, inhibited the thiram and ziram induced apoptotic cell death, indicating that thiram and ziram induce apoptotic neuronal cell death by Ca^2+ ^influx through non-selective cation channels [125].

The EBDCs maneb, mancozeb and metiram can induce malformations in rat foetuses, apparently mediated through formation of the ETU metabolite. The malformations predominantly affect the nervous system and the head, and they correspond to those expected as the result of thyroid insufficiency. They occur only at doses in excess of those that produce significant thyroid inhibition in adult rats, and they have been prevented, at least in part, by co-administration of thyroxin (reviewed in [126]). A key concern with thyroid inhibitors is that impaired thyroid function may alter hormone-mediated events during development, thereby possibly leading to permanent alterations in brain morphology and function [127,128]. Functional deficits are likely to occur during brain development even at mild degrees of hypothyroidism [129]. Even withing the normal range, a relatively slight reduction of the concentration of maternal thyroid hormones during pregnancy can lead to intelligence deficits in the children [130]. In addition to EBDCs/ETU, many other environmental contaminants have been found to interfere with thyroid function, for example the chlorophenoxy herbicide, 2,4-D (se below). Some of the mechanisms of action with respect to thyroid inhibition are shared by mancozeb/ETU and 2,4-D (including interference with uptake of iodide by the thyroid gland and interference with serum protein-bound iodide level) [131], and exposure to both EBDCs and chlorophenoxy herbicides may therefore result in additive effects.

Evidence that developmental exposure to maneb may be involved in development of Parkinson's disease (PD) later in life includes the finding that postnatal exposure of mice to maneb in combination with paraquat (a classic bipyridyl herbicide, which is no longer authorised in EU) led to a permanent and selective loss of dopaminergic neurons in the substantia nigra pars compacta [132]. The postnatal exposure to these pesticides enhanced the effect of the same pesticides administered during adulthood, relative to exposures during development only or adulthood only. Furthermore, exposure to maneb alone during gestation resulted in a dramatic response to paraquat in adulthood, including notable reductions in levels of dopamine and a loss of nigral dopamine neurons. Thus, these results support the notion that a silent neurotoxicity produced by developmental insults can be unmasked by insults later in life [132].

For specific dithiocarbamates, especially the EBDCs maneb and mancozeb, substantial evidence supports the possibility of developmental neurotoxicity. In addition, the likely mechanisms of toxicity for thiram and ziram indicate that these compounds too may be capable of causing developmental neurotoxicity in small doses.

### Chlorophenoxy herbicides

The chlorophenoxy herbicides are widely used for the control of broad-leaved weeds. Structurally, they consist of a simple aliphatic carboxylic acid moiety, which is attached to a chlorine- (or methyl-) substituted aromatic ring by an ether bond. *In vivo *the salts and esters are rapidly dissociated or hydrolysed, and therefore the toxicity of each chlorophenoxy compound depends principally on the acid form of the pesticide [133]. The chlorophenoxy herbicides bind strongly to albumin [134], and binding is favoured by longer acid chains and by more greatly substituted aromatic rings. Therefore the bioavailability and toxicity of the herbicides vary for different herbicides [135]. The mechanisms of neurotoxicity of the chlorophenoxy herbicides are incompletely known, but they seem to primarily involve cell membrane damage (reviewed in [135]).

2,4-Dichlorophenoxyacetic acid (2,4-D) is the most widely used chlorophenoxy herbicide and also the most widely studied. With respect to membrane damage, it does not cause significant penetration of lipid monolayers *in vitro *at concentrations below 0,1 μM [134], but at higher concentrations (10–100 μM) it increases bilayer width and causes deep structural perturbations of the hydrophobic region of model membrane systems. At the higher concentrations it also damages human erythrocyte cell membranes [136]. This dose- dependent effect on plasma membranes may in part explain the dose-dependent CNS toxicity caused by chlorophenoxy herbicides. In experimental animals (rats, mice and rabbits), only small amounts of herbicide were found in the brain following administration of 100 mg/kg or less [137-139], likely because low concentrations of herbicide have little effect on the plasma membranes comprising the blood-brain barrier. When exposing rats to high doses (250–500 mg/kg) of the herbicide, a reversible selective damage to the blood-brain barrier occurred, and as a result serum albumin and IgG could be detected in the brain along with the herbicide itself [140]. The severity of the herbicide-induced cerebrovascular damage in rats has been reported to increase in the order 2,4,5-T (2,4,5-trichlorophenoxyacetic acid) < MCPA (4-chloro-2-methylphenoxyacetic acid) < 2,4-D [141].

Chlorophenoxy herbicides can also disrupt cell membrane transport mechanisms. They competitively inhibit and ultimately saturate the organic anion transport system in the choroid plexus, which facilitates the removal of potentially toxic anions (including endogenous neurotransmitter metabolites and exogenous organic acids) [139,142,143]. Homovanillic acid and 5-hydroxy-3-indoleacetic acid, i.e. metabolites of dopamine and serotonin, respectively, accumulate in the CNS of rats following 2,4-D administration [144].

It has also been reported that 2,4-D induced neurotoxicity may be partly due to generation of free radicals. When incubating rat cerebellar granule cells with 2,4-D *in vitro*, glutathione (GSH) levels and catalase activity were significantly reduced, whereas generation of reactive oxygen species (ROS) and activity of selenium-glutathione peroxidase (Se-GPx) were augmented [145].

Furthermore, chlorophenoxy acids are structurally related to acetic acid and are able to form analogues of acetyl-CoA (e.g. 2,4-D-CoA) *in vitro*. Formation of such analogues has the potential of disrupting several pathways involving acetyl-CoA, including the synthesis of acetylcholine. Possible formation of choline esters (e.g. 2,4-D-Ach) may act as false cholinergic messengers (reviewed in [135]).

In cerebellar granule cells, 2,4-D produced a striking and dose-dependent inhibition of neurite extension, and *in vitro *2,4-D inhibited polymerisation of purified tubulin. Thus, it was suggested that at least one mechanism of 2,4-D neurotoxicity involves inhibition of microtubule assembly [146]. Yet another study with cerebellar granule cells showed that 2,4-D induced apoptosis when cells were exposed to millimolar concentrations of the compound [147].

Chlorophenoxy herbicide poisoning in humans is uncommon, but it may produce severe sequelae. In a review of 66 cases of chlorophenoxy herbicide poisoning [135], the majority of cases involved ingestion of 2,4-D, either alone or in combination with other chlorophenoxy herbicides. Neurotoxic effects included coma, hypertonia, hyperreflexia, ataxia, nystagmus, miosis, hallucinations, convulsions, fasciculations, and paralysis. Some degree of peripheral neuromuscular involvement occurred in approximately one third of the cases reviewed. Still, other constituents, such as surfactants or solvents, in the formulations of the herbicides could possibly have contributed to some of the effects observed [135].

The information with respect to possible neurological effects of chronic exposures to low doses of chlorophenoxy herbicides is sparse, and in a review from 2002, it was concluded that it is unlikely that 2,4-D has any neurotoxic potential at doses below those required to induce systemic toxicity [148]. However, a cohort study suggested an increased risk of amyotrophic lateral sclerosis (ALS) among workers chronically exposed to 2,4-D, compared to non-exposed employees at the same company, although this conclusion was based on only three deaths [149].

Although neurotoxicity in adults from low, chronic exposures to chlorophenoxy herbicides has not been reported, developmental exposure to low levels of these herbicides may still pose a threat. One case of cephalic malformations and severe mental retardation has been observed in an infant whose parents received prolonged exposure to 2,4-D via the dermal route from forest spraying [150].

Evidence of developmental neurotoxicity of chlorophenoxy herbicides, in particular 2,4-D, has also been obtained from experimental animals. For example, external treatment of fertilised hens' eggs with 2,4-dichlorophenoxyacetic butyl ester produced hypomyelination in the chicks, and reductions in "myelin markers" (including sulfatides, cerebrosides and 2'3'-cyclic nucleotide 3'-phosphohydrolase activity) were seen in chick embryos even before the period of active myelination [151]. A deficit in myelin lipid deposition was also detected in neonatal rats exposed to 2,4-D through lactation [152]. Other findings in response to neonatal exposure of rats to 2,4-D through lactation include a delay in CNS development [153], an increase in size and density of serotonin immunoreactive neuronal somata as well as an increase in fibre length in the dorsal and medial raphe nuclei [154]; and oxidative stress in specific brain areas, including midbrain, striatum, and prefrontal cortex [155].

Behavioural effects in the offspring have also been reported following prenatal and continued exposure to 2,4-D [156]. Also following prenatal and continued exposure of rats to 2,4-D, even beyond lactation, the dopamine D_2_-type receptor was increased about 40% in the striatum. Increased levels of the receptor were also found in the prefrontal cortex and cerebellum. However, when discontinuing exposure after weaning, no differences in dopamine D_2_-type receptors could be detected compared to control rats, suggesting that the effects of 2,4-D on these receptors may be reversible [157].

Thus, even though the evidence is sparse, some chlorophenoxy herbicides, in particular 2,4-D, have neurotoxic potentials and may cause developmental neurotoxicity.

### Bipyridyl herbicides

The bipyridyl herbicides share common toxic mechanisms [158,159]; paraquat has been used as a model substance, but is no longer allowed in the EU. Intracellularly, both paraquat and diquat undergo redox cycling, leading to the generation of superoxide anions. These anions may react to form hydrogen peroxide and subsequently the highly reactive hydroxyl radical, which may then cause lipid peroxidation and cell death [159,160]. Another factor contributing to toxicity is the depletion of nicotinamide adenine dinucleotide phosphate with a bound hydrogen ion (NADPH), as both herbicide redox cycling and hydrogen peroxide detoxification via glutathione are NADPH dependent [159,161]. In addition to redox-cycling, there is some evidence that paraquat may be able to interact with enzymatic targets in the CNS, such as AChE and butylcholinesterase [162].

The initial phase of moderate to severe intoxication with paraquat and diquat is characterised by renal and liver failure, but the subsequent clinical course differs between the two, with intestinal paralysis and fluid loss as prominent features of diquat intoxication [160,163-165]. In severe and usually fatal cases of diquat poisoning, coma has also been reported [160]. Severe neurological and neuropsychiatric complications due to brain stem infarction and/or intracranial haemorrhage have also been described [161,163,166].

In regard to long-term consequences of exposure to bipyridyl herbicides, paraquat is a prime suspect with respect to induction of PD. It causes selective degeneration of tyrosine hydroxylase immunopositive (TH^+^) neurons in the substantia nigra pars compacta, and long-term exposure has been found to increase the risk of PD in a Taiwan population that sprays paraquat on rice fields [167-169]. A case report has described PD following diquat exposure [170], but because of a long induction period and the difficulties in retrospective exposure assessment, the hypothesis of delayed appearance of degenerative nervous system disease is difficult to verify. Since both paraquat and diquat can generate the formation of ROS, these compounds may well be involved in neurodegenerative diseases other than PD, such as Alzheimer's disease, but little evidence is available to evaluate this potential.

Even though it is rather clear that the cytotoxicity of paraquat involves oxidative stress [171], the sensitivity of dopaminergic neurons is difficult to explain [172]. Possibly, the dopaminergic neurons may be particularly sensitive to the reactive oxygen species (ROS) from paraquat, since dopamine metabolism also creates ROS [173]. In mice treated with paraquat once a week for 3 weeks, the effect on catecholaminergic neurons was reminiscent of that in PD, with a preferential loss of dopaminergic neurons in the substantia nigra pars compacta. This is consistent with the results from several similar studies [168,169,171].

PD has also been explored as a relevant outcome with respect to developmental neurotoxicity. When neonatal mice were exposed to paraquat, a marked hypoactive condition was apparent at 60 days of age and became even more pronounced at 120 days of age [174]. Furthermore, paraquat reduced the striatal content of dopamine and metabolites without affecting serotonin [174]. As already mentioned above under dithiocarbamates, other evidence suggests that maneb and paraquat may jointly and individually induce loss of dopaminergic neurons in mice. Administration of these pesticides postnatally enhanced the effect of the same pesticides administered during adulthood. Furthermore, exposure to maneb alone during gestation resulted in a dramatically increased response to paraquat in adulthood, including notable reductions in levels of dopamine and a loss of nigral dopamine neurons [132]. Similarly, the greatest effect on locomotor activity in mice occurred in males after exposure to maneb prenatally and to paraquat in adulthood [175]. This finding was supported by decreased levels of striatal dopamine, increased striatal dopamine turnover, and selective reduction in tyrosine hydroxylase-immunoreactive neurons of the substantia nigra pars compacta.

These observations are in agreement with the notion that an initially silent toxicity was later unmasked, and was affected by the specific order-of-presentation of the pesticides in regard to the developmental stage (not just an effect of the combination of pesticides). Thus, it seems that prenatal exposure to maneb, rather than paraquat, may sensitise/predispose mice to development of PD (or lead to a state of increased vulnerability), whereas paraquat exposure later in life may unmask the silent toxic effect of the earlier maneb exposure and then lead to clinical symptoms of the disease. Therefore it is possible that in the case of PD, developmental exposure to paraquat may not be as damaging as later exposure, particularly if this later exposure follows developmental exposure to maneb.

### Fumigants

The mechanisms of toxicity employed by various types of fumigants are poorly known. A common mechanism of action is not expected, and the fumigants are therefore reviewed one by one.

Among metal phosphide fumigants, aluminium phosphide is one of the most extensively used. The phosphides are very toxic, because of their ability to liberate phosphine under moist conditions (reviewed in [176]). Phosphine is a reductant and predictably reacts with metal ions such as the iron in haem and the divalent metals of metal dependent enzymes [177]. Cytochrome c oxidase, of the mitochondrial electron transport chain, has been suggested as the primary site of action for phosphine [176,178,179]. A 50% inhibition of this enzyme was found to be sufficient for generation of superoxide anions, and it was suggested that the toxicity of phosphine was due to damage by free radicals [178]. In agreement with this hypothesis, aluminium phosphide has been found to increase lipid peroxidation in rat brain [180].

Further, in 45 phosphine poisoning patients, increased levels of superoxide dismutase (SOD) and malondialdehyde (MDA) were detected in non-survivors, while catalase was inhibited [181]. Oxidation of phosphine can lead to formation of reactive phosphorylating species [182], thus suggesting that effects on cholinesterase may be possible [183]. Studies of grain fumigant applicators [184] and *in vitro *studies of human red blood cells [185] have shown that significant phosphine-induced inhibition of red blood cell cholinesterase occurs at concentrations of phosphine exceeding 10 μg/ml.

Neurological changes like ataxia, stupor, tremors and convulsions have been observed following aluminium phosphide poisoning. Acute hypoxic encephalopathy has also been observed following aluminium phosphide poisoning, which may lead to death as a result of complete depression of the central nervous system and paralysis of the respiratory centres of the brain (reviewed in [176]).

With respect to consequences of chronic phosphide exposure knowledge is sparse, but one descriptive study reported that most of a group of workers exposed to zinc phosphide had one or more neuropsychiatric symptoms including anxiety, impotence and easy fatigue. About half of the workers showed hyperreflexia, polyneuropathy, lumbar radiculopathy, and cervical myelopathy, as well as anxious mood, impaired attention, and psychomotor stimulation. EEG recordings showed abnormal findings in 17.4% of the subjects, mainly those with longer exposure [186]. These preliminary findings should invite further studies in this area.

For the fumigant sulfuryl fluoride, very little is known concerning the mechanism of toxicity. The fluoride ion may play a role, since many of the observations in rodents overexposed to sulfuryl fluoride are typical of acute fluoride poisoning [187]. In humans, short-term inhalation exposure to high concentrations of sulfuryl fluoride has been reported to cause central nervous system effects [188]. A case report describes an elderly couple, who returned to their home 5–8 hours after fumigation with sulfuryl fluoride. The wife experienced weakness, nausea, and repeated vomiting, while the husband complained of dyspnea and restlessness. Within 48 hours the husband had a generalised seizure followed by cardiopulmonary arrest. The wife died within 7 days due to ventricular fibrillation. The serum fluoride concentration of the wife six days after the fumigation was reported to be as high as 0.5 mg/L [189].

Workers with a chronic, low level exposure to sulfuryl fluoride showed non-significantly reduced performance on all applied neurobehavioural tests compared to the control group in one study [190]. Education levels, ethnicity and drug use differed between the workers and the control group in this study. In a later study of structural fumigation workers [191], sulfuryl fluoride exposure during the year preceding the examination was associated with significantly reduced performance on the Pattern Memory Test (a test of cognitive and visual memory) and an olfactory test. No pattern of cognitive deficits was detected.

None of these fumigants has been examined in detail for possible developmental neurotoxicity. Pregnant rats and rabbits exposed to sulfuryl fluoride were reported to show no evidence of embryotoxicity, foetotoxicity, or teratogenicity at concentrations of sulfuryl fluoride as high as 225 ppm, although body weights of rabbit foetuses as well as the dams at the highest exposure were lower than in the control group [192].

In regard to phosphine, a large epidemiological study found that adverse neurological and neurobehavioural developmental effects clustered among children fathered by applicators of phosphine (odds ratio = 2,48; 95% confidence interval: 1.2, 5.1) [193]. Other than this study, no information regarding developmental neurotoxicity of phosphine was identified.

### Other pesticides

The present review on neurotoxicity has focused on a small number of substances out of the total number approved for use as pesticides in the EU. Quite likely, much evidence exists on neurotoxicity, but has not been published in biomedical journals. Nicotine, warfarin and ethanol are additional well documented neurotoxicants, but their primary use is not as pesticides. The same applies to other substances listed, such as sodium hypochlorite and aluminium sulfate, which may potentially add to neurotoxic hazards.

### Public health implications

Some of the substances belonging to the groups of pesticides reviewed here (including OPs, carbamates, pyrethroids, ethylenebisdithiocarbamates, chlorophenoxy herbicides, and bipyridyl herbicides) appear to share common mechanisms of action with respect to induction of neurotoxicity. Thus, members of these chemical groups of pesticides other than those identified as neurotoxic in the present review, would then be highly likely also to cause neurotoxicity. For other groups of pesticides without a plausible common mechanism of action (e.g. the fumigants), it is not possible to predict whether group members might share neurotoxicity potentials.

Further refinement of this prediction is difficult. As anticipated, the literature on developmental neurotoxicity is sparse for most of the pesticides. However, some evidence does exist to suggest that several of the neurotoxic pesticides in current use in the EU may cause developmental neurotoxicity in small doses. Table 2 summarises the existing evidence of developmental neurotoxicity for groups of pesticides with common mechanisms of action.

**Table 2:** Evidence of developmental neurotoxicity caused by pesticides belonging to groups with likely common mechanisms of neurotoxicity

Group of pesticides (n)*	**Mechanism of neurotoxicity**	**Developmental neurotoxicity reported in humans**	**References**	**Developmental neurotoxicity reported in animals**	**References**
Organophosphates (8)	Inhibition of AChE (+ interference with signaling cascades at low doses)	Reflex abnormalities in neonates + affected mental development	[7,8]	Altered programming of synaptic development in rats (Chlorpyrifos)	[50,51]
		
		Reduced head circumference in infants + anomalies in primitive reflexes (Chlorpyrifos)	[61,9]	Behavioural abnormalities including changes in locomotor skills and cognitive performance in rats and mice (Chlorpyrifos)	[43-46]
		
		Reduced birth weight and length + developmental delay at 3 years of age (Chlorpyrifos)	[62,10]		
		
		Visuospatial deficits (prenatal exposure) + increased reaction time (current exposure in children)	[11]		
		
		Reduced short term memory and attention (Methyl parathion)	[12]		

Carbamates (5)	Inhibition of AChE (+ oxidative stress)	No reports were found		No reports were found	

Pyrethroids (7)	Prolongation of kinetics of voltage-gated sodium channels			Increased motor activity, lack of habituation, changes in mAChR density in mice	[85-88]
				
				Learning changes in rats	[89]
				
				Changes in motor activity in rats	[90]
				
				Changes in sexual behaviour and higher activity of the dopaminergic system in rats	[91]
				
				Changes in mAChR expression in rats	[92,93]
				
				Changes in blood-brain permeability in rats	[94]
				
				Affected development of reflexes, swimming ability, open field activity in mice (parental exposure prior to mating)	[96]

Dithiocarbamates (EBDCs) (6)	Generation of ROS (metal chelating capacity, uncoupling of mitochondrial electron transport chain) The EBDC metabolite, ETU, inhibits thyroid peroxidase (synthesis of thyroid hormones)			Maneb (in combination with paraquat) induces loss of dopaminergic neurons in substantia nigra pars compacta in mice	[132]
				
				The metabolite of EBDCs, ETU, induces malformations of the nervous system (corresponding to thyroid insufficiency) in rats	Reviewed in [126]

Chlorophenoxy herbicides (11)	Not completely known: includes membrane damage, generation of free radicals, perhaps uncoupling of oxidative phosphorylation	A case of cephalic malformations and severe mental retardation in infant whose parents were heavily exposed to 2,4-D	[150]	Hypomyelination in chicks (2,4-D)	[151]
				
				Deficit in myelin lipid deposition in rats (2,4-D)	[152]
				
				Delayed CNS development in rats (2,4-D)	[153]
				
				Increased size and densitiy of serotonin-reactive neuronal somata and increased fiber length in dorsal and medial raphe nuclei in rats (2,4-D)	[154]
				
				Oxidative stress in specific brain areas (midbrain, striatum, prefrontal cortex) in rats (2,4-D)	[155]
				
				Behavioural effects in rats including delay of righting reflex, negative geotaxis + motor abnormalities, excessive grooming and vertical head movements, hyperactivity (2,4-D)	[156]

Bipyridyl herbicides (1)	Induction of oxidative stress			Involvement of developmental exposure to paraquat in later development of PD like features in mice	[174]
				
				Paraquat (in combination with maneb) induces loss of dopaminergic neurons in substantia nigra pars compacta in mice	[132]

*The number in parenthesis is the total number of pesticides from each group currently authorised for use in the EU as of August 2008. Only major evidence on developmental neurotoxicity in humans or in laboratory animals has been included.

Most evidence is available for the OPs, especially chlorpyrifos. The evidence strongly supports the notion that developmental neurotoxicity may be induced by very low exposure levels, i.e. much below those causing any neurotoxicity in adults. Most evidence still comes from studies in laboratory animals, but some epidemiological data are highly suggestive of neurotoxic effects caused by developmental exposure of humans to OPs (including chlorpyrifos). In the case of OPs, which share inhibition of AChE as a common mechanism of action in high doses, chlorpyrifos may employ other mechanisms of action at lower doses associated with developmental neurotoxicity. In fact developmental neurotoxicity in mice and rats can be induced at doses, which cause no detectable inhibition AChE [43-46,51]. Thus, even though a group of pesticides shares a common mechanism of action at larger doses, it cannot be excluded that compound specific mechanisms may also exist at lower doses. This fact unfortunately complicates the extrapolation of developmental neurotoxicity from one member of a group of pesticides to another. However, the combined human evidence on developmental neurotoxicity associated with OP exposure cannot be ascribed to chlorpyrifos alone.

Other than for OPs, the evidence of developmental neurotoxicity in humans is sparse, but evidence on developmental neurotoxicity in laboratory animals exists for pyrethroids, ethylenebisdithiocarbamates, and chlorophenoxy herbicides (mainly 2,4-D).

In the case of dithiocarbamates, evidence from laboratory animals suggests that developmental exposure to, e.g. maneb, may predispose the individual to development of PD later in life in response to another exposure, in particular paraquat. Other experimental studies suggest that prenatal exposure to paraquat can also predispose to development of PD later in life. It seems that the greatest effect of paraquat with respect to induction of PD is obtained from exposure later in life, following early priming exposure to maneb [175]. Although PD is a degenerative disease associated with aging, these data suggest that developmental exposure to pesticides (e.g. maneb) may constitute an aetiological factor that sensitises the individual to later insults (e.g. subsequent pesticide exposure, and aging).

For the remaining pesticides that belong to groups without a common mechanism of toxicity, the lack of research on developmental neurotoxicity complicates the evaluation of their safety. In a few cases (e.g. the fumigant sulfuryl fluoride), the existing evidence from animal experiments indicates that developmental neurotoxicity may be unlikely to occur at doses below those causing maternal toxicity [192,194]. Still, in these experiments, possible later emerging effects or sensitisation caused by developmental exposure has not been studied, so any conclusion in this regard would be tentative.

On the other hand, with respect to the metal phosphide fumigants, which release phosphine under moist conditions, some evidence of developmental neurotoxicity does exist. An epidemiologic study has found adverse neurological and neurodevelopmental effects among children fathered by applicators of phosphine [193]. For the remaining pesticides reviewed, no data from either human or animal studies could be located by our search.

This review has focused on those pesticides, for which human neurotoxicity has been reported in relation to specific exposures to the particular pesticide. This means that we have excluded poisoning cases involving more than one compound, where the contribution by each substance may be unknown. Thus, our list of neurotoxic pesticides is likely a substantial underestimate of the true number of neurotoxic pesticides. The fact that no poisoning incident with neurotoxic effects has been reported for a given pesticide is of course no guarantee that the pesticide is not neurotoxic, especially in regard to developmental exposure. A prudent evaluation of the evidence would therefore suggest that, if individual members of a chemical grouping of pesticides have been documented as neurotoxic, then all members of that group should be considered to be neurotoxic as well.

In addition to the problem of scarce – in many cases even non-existing – scientific evidence on developmental neurotoxicity of the pesticides in current use, some discrepancies exist between results of animal studies. An important factor in regard to apparent discrepancies is that the timing of exposure varies between studies. In some studies, animals are exposed prenatally, in other studies neonatally (during the first weeks of life), and in some studies both prenatally and neonatally. The timing of exposure may greatly influence the extent and type of neurotoxicity induced. Most animal studies have been performed in rodents, where brain development is mainly neonatal and spans the first three to four weeks of postnatal life [14,195]. Thus, although neurotoxic effects may be induced in rodents by only prenatal exposure, it is highly likely that these studies underestimate the neurotoxic effects, which may occur in response to prenatal exposure of humans, where the third trimester of pregnancy is a crucial period of brain development.

A further concern is that humans are very likely to be exposed to a number of pesticides and other neurotoxic compounds simultaneously. Because it is possible that some of these may have synergistic or additive effects, exposure to even very low doses during development may cause neurotoxic damage.

In addition to "direct" neurotoxicity, there is also evidence that several pesticides may indirectly cause neurotoxicity, e.g. by interference with thyroid function. Some 60% of all herbicides, in particular 2,4-D, acetochlor, aminotriazole, amitrole, bromoxynil, pendamethalin, and thioureas have been reported to interfere with thyroid function (reviewed in[196]). In addition, EBDC dithiocarbamates, organophosphates and synthetic pyrethroids are thought to interfere with thyroid function (reviewed in [197]). A key concern with thyroid inhibitors is that impaired thyroid function may alter hormone-mediated events during development, leading to permanent alterations in brain morphology and function [127,128]. Other types of endocrine disruption can conceivably lead to neurobehavioural deficits, but this evidence has not been included here.

The current evidence can therefore be summed up as follows. A substantial proportion of pesticides in current use are known to be neurotoxic. However, neurotoxicity potentials of pesticides have not necessarily been examined, as legally mandated tests do not require specific assessment of neurotoxic potentials, apart from tests for peripheral neurotoxicity in hens required for OPs. A test battery for developmental neurotoxicity has only recently been completed by OECD, and very limited test data are available for pesticides. Because developmental neurotoxicity can occur at exposures much below those that cause toxicity to the adult brain, usage restrictions and legal limits for pesticide residues in food may not be sufficiently protective against developmental neurotoxicity. In addition, experimental, clinical and epidemiologic evidence supports the notion that neurotoxicity may be much more severe and possibly irreversible when the exposure occurs during early development.

Unless documentation exists for a particular pesticide to falsify this notion, all neurotoxic pesticides should be considered likely of inducing developmental neurotoxicity at low doses. The public health significance of this issue is illustrated by the epidemiologic observation of neurodevelopmental deficits at exposure levels that seem to be commonly occurring in the general population. Although the exact identity of the causative substances may be uncertain, pesticide contamination of foods is common in the EU, it often exceeds previously identified legal limits, and it involves substances that are known to be neurotoxic. Given the substantial impact of neurodevelopmental abnormalities in society and the likely impact of environmental aetiologies, prevention of pesticide exposure appears to be an obvious public health priority.

## Conclusion

Given the widespread use and exposure to pesticides, the general lack of data on developmental neurotoxicity is a serious impediment. For certain pesticides, a requirement exists for neurotoxicity tests in adult animals, but developmental neurotoxicity is usually not considered when determining pesticide safety. Experimental, clinical, and epidemiologic evidence suggests that neurotoxic pesticides can also cause developmental neurotoxicity, and that the effects are more severe and lasting, and that they occur at much lower exposure levels. Some of this evidence relates to model substances that have now been banned or restricted, but currently used substances with similar mechanisms of toxicity should be regarded to share the same toxic potentials. Thus, many widely used pesticides, such as organophosphates, carbamates, pyrethroids, ethylenebisdithiocarbamates, and chlorophenoxy herbicides should be considered neurodevelopmental toxicants, unless convincing evidence exists for individual substances that they deviate from the general group characteristics. Given the likely environmental aetiology of neurodevelopmental deficits and their importance to families and to society, prevention of exposures to neurotoxic pesticides should be made a public health priority. Existing uncertainties should not be used as an excuse for rejecting precautionary action.

## Abbreviations

ACh: Acetylcholine; AChE: Acetylcholinesterase; ADHD: Attention Deficit Hyperactivity Disorder; AMP: Adenosine monophosphate; ALS: Amyotrophic Lateral Sclerosis; CNS: Central Nervous System; CREB Ca^2+^/cAMP Response Element Binding protein; CT: Computed Tomography; 2,4-D: 2,4-Dichlorophenoxyacetic acid; DEDC: Diethyldithiocarbamate; EBDC: Ethylenebisdithiocarbamate; EEG: Electroencephalogram; ETU: Ethylenethiourea; EU: European Union; GABA: Gamma-aminobutyric acid; GSH: Glutathione; HSDB: Hazardous Substances Data Bank; IgG: Immunoglobulin G; mAChR: muscarinic acetylcholine receptor; MCPA: 4-chloro-2-methylphenoxyacetic acid; MDA: Malondialdehyde; MPTP: 1-methyl-4-phenyl-1,2,3,6-tetrahydropyridine; MRI: Magnetic Resonance Imaging; NADPH: Nicotinamide Adenine Dinucleotide Phosphate with a bound Hydrogen ion; NIOSH: National Institute of Occupational Safety and Health; NTE: Neuropathy Target Esterase; OECD: Organisation for Economic Co-operation and Development; OP: Organophosphate; OPIDP: OrganoPhosphate-Induced Delayed Polyneuropathy; PC12 cells: Cancer cell line from a pheochromocytoma of the rat adrenal medulla; ROS: Reactive Oxygen Species; Se-GPx: Selenium-glutathione peroxidase; SOD: Superoxide dismutase; 2,4,5-T: 2,4,5-trichlorophenoxyacetic acid; TH^+^: Tyrosine Hydroxylase immunopositive; TPX: Thyroid peroxidase.

## Competing interests

PG is an editor of Environmental Health but was not involved in the editorial handling of this manuscript. The authors declare that they have no competing interests.

## Authors' contributions

MBP, HRA and PG jointly conceived the review, MBP and HRA from mechanistic and toxicologic considerations and PG from an epidemiologic viewpoint. MBP conducted the literature survey and wrote the first draft, which all authors revised and updated. The final manuscript was approved by all authors.
